# Impact of fatigue on work productivity and health-related job loss

**DOI:** 10.1093/occmed/kqae056

**Published:** 2024-07-06

**Authors:** G J Macfarlane, S D’Angelo, G Ntani, K Walker-Bone

**Affiliations:** Medical Research Council/Versus Arthritis Centre for Musculoskeletal Health and Work, School of Medicine, Medical Sciences and Nutrition, University of Aberdeen, Aberdeen, AB25 2ZD, UK; Medical Research Council/Versus Arthritis Centre for Musculoskeletal Health and Work, University of Southampton, Southampton, SO16 6YD, UK; Medical Research Council/Versus Arthritis Centre for Musculoskeletal Health and Work, University of Southampton, Southampton, SO16 6YD, UK; Medical Research Council/Versus Arthritis Centre for Musculoskeletal Health and Work, University of Southampton, Southampton, SO16 6YD, UK; Medical Research Council/Versus Arthritis Centre for Musculoskeletal Health and Work, University of Southampton, Southampton, SO16 6YD, UK; Monash Centre for Occupational & Environmental Health, Monash University, Melbourne, Victoria, VIC 3004, Australia

## Abstract

**Background:**

Fatigue is commonly reported in population surveys and has been identified in patients with health conditions as a key co-morbidity which makes remaining in work challenging. Such patients, however, rarely have access to programmes to help them manage their fatigue.

**Aims:**

To quantify the relationship between fatigue, work impairment and health-related job loss.

**Methods:**

We use data from the Health and Employment After Fifty study, a longitudinal study of people aged 50–64 years when recruited through general practices in England in 2013–14. During follow-up, fatigue was measured using the Fatigue Assessment Scale, work impairment was assessed using the Work Productivity and Activity Impairment scale, and changes in employment status were recorded.

**Results:**

A total of 2743 participants were eligible for the current analysis; 23% satisfied criteria for being fatigued. People who were fatigued were less likely to have a partner, university degree, be physically active and were more likely to be obese. Their job was more likely to involve shifts, be perceived as insecure, have reported difficulties coping with job demands, and be unsatisfying. After adjustment for socio-economic, lifestyle and work-related factors, they were almost twice as likely to report both work impairment (relative risk 1.8; 95% confidence interval [CI] 1.6, 2.1) and future health-related job loss, although the latter effect was only in those with other morbidities (incidence rate ratio 1.96; 95% CI 1.03–3.72).

**Conclusions:**

Providing evidence-based support for workers with health conditions who experience fatigue may have an important impact at a population level in terms of extending working lives.

## Teaser

Fatigue is a common co-morbidity for a range of health conditions and is identified by patients as an important factor preventing them from staying at work. In this longitudinal study of middle-aged people in England, fatigue doubles the risk of being impaired in doing your job and of leaving work for health reasons. Evidence-based programmes to support the management of fatigue should be available to workers with health conditions.

**Key learning points**

**What is already known about this subject:**

- Patients identify fatigue (in addition to pain) as key symptoms that make it challenging to remain working with a health condition.
- Whether fatigue independently predicts job loss is not clear.

**What this study adds:**

- Fatigue, in the context of other health conditions, approximately doubles the risk of health having an impact on productivity and leaving work due to ill health.
- The role of fatigue is independent of other factors.
- Taken together with data from qualitative studies and randomized controlled interventions, fatigue is likely a causal factor in leaving work due to ill health.

**What impact this may have on practice or policy:**

- People who are working, with fatigue in the context of a health condition, should be given access to evidence-based management for their symptoms to reduce the chance of having to leave work due to ill health.

## Introduction

Fatigue is a commonly reported symptom, and the annual incidence of fatigue (as a symptom presenting to primary care in the UK) has been estimated at ~1500 per 100 000 [1]. Fatigue refers not to the tiredness from physical or mental efforts but an overwhelming sense of exhaustion and the onset of which is either unpredictable or out of proportion to exertions. Fatigue is a key symptom impacting quality of life in the context of many long-term health conditions [2].

In a study of patients receiving biologic therapy for psoriatic arthritis (PsA) across 13 countries, 68% reported moderate or severe fatigue and demonstrated significantly worse scores for quality of life and work participation [3]. In the RA-BEAM trial for the management of rheumatoid arthritis, improvements in work productivity with active treatments were shown to be mediated through reductions in disease activity, fatigue and pain [4]. In relation to the ArthritisPower registry in the USA, patients with conditions across the musculoskeletal spectrum identified fatigue as a key priority for tracking in relation to assessing disease management [5]. Patients with other inflammatory conditions, such as inflammatory bowel disease (IBD), identify fatigue as the most common reason for work absence [6], while a systematic review amongst cancer survivors identified fatigue as a key factor in work outcomes [7].

International data have shown large numbers of people became economically inactive during the coronavirus disease 2019 (COVID-19) pandemic [8]. While these changes have now reversed in most countries, some, including the UK, still have higher numbers of people economically inactive compared with the time before the pandemic, and this has important economic consequences [9]. Ill health is believed to be an important reason for these trends, particularly amongst older workers. Indeed, one of the conditions thought to contribute to the rise in economic inactivity in the UK is ‘long COVID’, which has fatigue as a key symptom [10]. If we are to increase the number of people working, we must address important health issues which have caused people to leave work.

In this context, our aim is to quantify the role of fatigue in work impairment and health-related job loss (HRJL): we will address four specific research questions: (1) What socio-demographic, lifestyle and work-related factors are related to fatigue? (2) Is fatigue associated with reduced work productivity? (3) Is fatigue predictive of HRJL after adjustment for potentially confounding factors? (4) Do people with recent HRJL report higher levels of fatigue than those who have continued working?

## Methods

The Health and Employment After Fifty (HEAF) cohort study was established in 2013–14. People registered at 24 general practices in England (all within the Clinical Practice Research Database) were invited to take part (*n* = 35 359), and 8134 people aged 50–64 years returned a baseline questionnaire. People were eligible to participate regardless of their employment status. At and since recruitment, information has been collected by annual postal questionnaire on demographic characteristics, work status, job characteristics, social and financial circumstances, retirement plans, and mental and physical health. The detailed methodology has been published elsewhere [11]. At baseline and each annual follow-up, participants were asked about self-reported health and whether their employment status had changed since last completing a HEAF questionnaire. Those reporting a job change or exit were asked to report the dates of the change, and whether a health condition was at least partly responsible for leaving each job, which was then defined as HRJL.

Fatigue was assessed at the 4-year follow-up using the Fatigue Assessment Scale (FAS) which was developed in large samples of the Dutch working and general population [12]. Based on factor analyses, the FAS is considered unidimensional, and consequently, only a total score is calculated. The instruction of the FAS is directed at how a person usually feels. FAS is a 10-item self-report scale and each of its 10 questions has five possible answers ranging from ‘Never’ (scored 1) to ‘Always’ (scored 5). Scores from each question are summed up resulting in a total fatigue score ranging from 10 to 50. We have used both a binary classification with FAS scores >22 being indicative of fatigue, as reported by Michielsen *et al*. [13], and a continuous measure. Respondents were asked about whether they had seen a doctor for any of 16 health problems, sleep problems in the past 3 months assessed through a four-part question based on the Jenkins sleep questionnaire [14], and about sites of body pain experienced during the past month.

Impairment while working due to health was used as a measure of work productivity and was assessed at 4 years follow-up using the Work Productivity and Activity Impairment Questionnaire (General Health version) [15]. Work impairment due to health was expressed as a per cent (range: 0–100) and was then dichotomized with those scoring >0 characterized as ‘impaired’. The physical work activity score was computed according to how many of six activities the person performs on an average working day (kneeling, climbing a ladder, digging, lifting, standing for 3 hours at a time, hard physical work) with a score range from 0 to 6. Body mass index was computed with self-reported height and weight.

Baseline socio-demographics, lifestyle and work characteristics reported at 4 years follow-up for participants with and without fatigue were described and compared with Pearson Chi-squared test (Research Question 1). Poisson regression modelling with robust standard errors was used to explore: the cross-sectional association between fatigue and work impairment (expressed in its dichotomous form: ‘impaired’ versus ‘not impaired’) due to health at 4 years follow-up (Research Question 2), the longitudinal association between fatigue at 4 years follow-up and HRJL (‘yes’ versus ‘no’) assessed approximately a year later (Research Question 3); the impact of HRJL between 3- and 4 years follow-up on fatigue (used in its dichotomous form: ‘fatigued’ versus ‘not fatigued’) assessed at 4 years follow-up (Research Question 4). Estimates on the cross-sectional associations were expressed as relative risks (RRs) and those of the longitudinal associations as incidence rate ratios (IRRs) with 95% corresponding confidence intervals (CIs). All effects were first presented unadjusted and then adjusted for all socio-demographic, lifestyle and work-related factors.

Analyses were performed with a statistical software Stata v17.0. Ethical approval was issued from the National Health Service Research Ethics Committee Northwest—Liverpool East (Reference 12/NW/0500), and all participants gave written informed consent.

## Results

A total of 8134 people (55% women) aged 50–64 years agreed to participate in HEAF at baseline (2013–14). Sixty-eight per cent of participants originally recruited were employed or self-employed, 84% owned their home with/without a mortgage and 70% had a spouse/partner. A third had a university degree or a higher professional qualification, while a similar proportion (36%) had no qualification.

At 4 years follow-up (in 2017–18), 5791 participated; these included 2958 workers, 130 of whom had left job on health grounds (HRJL) and 2703 who had left job for reasons other than health. For Research Questions 1 and 2, we used the full sample of workers at 4 years follow-up, who provided usable data on fatigue and job productivity. For Research Question 3, we included those who were successfully followed up a year later. Finally, for Research Question 4, at 4 years follow-up, we compared those who were still working with those who had exited the workforce for health reasons between 3- and 4-year follow-up.

What socio-demographic and work factors are related to fatigue? A total of 2743 participants were included in this analysis, of whom 23% satisfied the criteria for being fatigued. Comparing those fatigued and not fatigued according to information provided at baseline, those fatigued were slightly younger (55.9 versus 56.7 years) and exhibited striking baseline socio-economic differences (Table 1). In the fatigued group, 8% more were single/widowed/divorced, while 7% more had no educational qualifications. There were also considerable differences in lifestyle factors: people with fatigue, were less likely to report undertaking any physical activity (69% versus 79%), or to have weekly contact with family/friends (84% versus 88%) but were more likely to be a current or ex-smoker (46% versus 41%), to drink no or little alcohol (26% versus 17%), and to be obese (30% versus 20%). Comparing work-related factors reported at Follow-up 4 (Table 2), those fatigued were more likely to have reported a job that often involved rotating shifts (18% versus 12%), work that they perceived to be insecure (61% versus 42%), for which they had difficulty coping with the physical (62% versus 22%) or mental demands (66% versus 25%), and with which they were more likely to be dissatisfied or very dissatisfied (16% versus 4%).

**Table 1.** Socio-demographic (assessed at baseline) and fatigue (assessed at 4-year follow-up)

	Not fatigued (*n* = 2125) *n* (%)	Fatigued (*n* = 618) *n* (%)	*P* value
Socio-demographic			
Marital status			
Married/civil partnership	1530 (72)	394 (64)	***
Single/widowed/divorced	580 (27)	218 (35)	
Missing	15 (1)	6 (1)	
Highest educational qualification			
No qualifications/school	614 (29)	223 (36)	***
Vocational training certificate	657 (31)	222 (36)	
University degree/higher	854 (40)	173 (28)	
Housing tenure			
Owned outright	994 (47)	238 (39)	***
Mortgaged	912 (43)	251 (41)	
Rented/rent free	182 (9)	113 (18)	
Missing	37 (1)	16 (3)	
How are you managing financially?			
At least getting by	1975 (93)	501 (81)	***
Managing with difficulty	113 (5)	103 (17)	
Missing	37 (2)	14 (2)	
Lifestyle	*N(%)*	N(%)	
Weekly physical activity			
Some	1680 (79)	426 (69)	***
None	285 (13)	131 (21)	
Missing	160 (8)	61 (10)	
Weekly contact with friends/family not in your household			
Some	1871 (88)	516 (83)	**
None	134 (6)	60 (10)	
Missing	120 (6)	42 (7)	
Obesity (body mass index)			
Underweight (<18 kg/m^2^)	12 (1)	4 (1)	***
Normal (18.5–24.9 kg/m^2^)	807 (38)	168 (27)	
Overweight (25–29.9 kg/m^2^)	826 (39)	247 (40)	
Obese (≥ 30 kg/m^2^)	425 (20)	183 (30)	
Missing	55 (3)	16 (3)	
Alcohol intake per week			
Low/no drinker (≤1 unit pwk)	358 (17)	158 (26)	***
Moderate (2–14 units pwk)	1209 (57)	298 (48)	
Heavy (15+ units pwk)	421 (20)	97 (16)	
Missing	137 (6)	65 (11)	
Smoking status			
Never	1238 (58)	328 (53)	NS
Ex/current	872 (41)	284 (46)	
Missing	15 (1)	6 (1)	

**P < 0.01; ***P < 0.001; NS: not significant.

**Table 2.** Work-related factors (assessed at baseline) and fatigue (assessed at 4-year follow-up) among those at work at baseline

	Not fatigued (*n* = 2061) *n* (%)	Fatigued (*n* = 599) *n* (%)	*P*
Employment contract			
Permanent	1569 (76)	480 (80)	NS
Temporary/renewable	112 (5)	36 (6)	
Self-employed	352 (17)	77 (13)	
Missing	28 (1)	6 (1)	
Job involves rotating/variable shifts			
Sometimes/rarely/never	1743 (85)	474 (79)	**
Often	280 (14)	115 (19)	
Missing	38 (2)	10 (2)	
Job involves night work			
Sometimes/rarely/never	1942 (94)	547 (91)	**
Often	89 (4)	46 (8)	
Missing	30 (2)	6 (1)	
Physical work activity score (median [IQR])	0 (0-2)	1 (0–3)	***
Job satisfaction			
Very satisfied/satisfied	1947 (95)	528 (88)	***
Dissatisfied/very dissatisfied	91 (4)	66 (11)	
Missing	23 (1)	5 (1)	
Job security			
Secure when well or ill	1096 (53)	240 (40)	***
Insecure when well or ill	942 (46)	353 (59)	
Missing	23 (1)	6 (1)	
Currently coping with physical demands of the job			
Easily	1676 (81)	300 (50)	***
Some difficulty or more	363 (18)	294 (49)	
Missing	22 (1)	5 (1)	
Currently coping with mental demands of the job			
Easily	1553 (75)	296 (49)	***
Some difficulty or more	486 (24)	296 (49)	
Missing	22 (1)	7 (1)	

**P < 0.01; ***P < 0.001; NS: not significant.

Is fatigue associated with reduced work productivity? Amongst the 2743 included in the analysis, there was a marked difference in work impairment: of those with fatigue, 55% reported health-related impairment to productivity compared to 19% in those without fatigue. In further analyses, impairment was dichotomized into ‘none’ or ‘some’: amongst those not work impaired, 14% were fatigued, as compared with 46% who reported being work impaired. The crude RR of work impairment in those fatigued as compared with those not fatigued was 2.9 (95% CI 2.6, 3.3). Indeed, the relationship was robust to further adjustment for all socio-demographic and work-related factors listed in Tables 1 and 2, respectively (adjusted RR 1.8; 95% CI 1.6, 2.1). Regression analysis showed that for every point increase in the fatigue score, the risk of work impairment increased by 9% (RR 1.09; 95% CI 1.08–1.09), and this was also unaffected by adjustments.

Does fatigue predict HRJL? Of the 2632 participants included in this analysis, HRJL between 4 and 5 years follow-up was reported by 5% (*n* = 116) participants. As a binary variable, there was a significant association of fatigue with future HRJL in the crude model (IRR 3.12; 95% CI 2.20, 4.44); however, associations weakened when fully adjusted (IRR 1.47; 95% CI 0.94, 2.30). We undertook some sensitivity analyses to first determine whether the relationship with HRJL was present both in those with and without reported sleep problems and there was no substantial difference, in fully adjusted models when run separately for these groups. Second, we wished to determine whether the effect of fatigue was present in those with and without other morbidities. When separate fully adjusted models were run, the effect of fatigue in HRJL was seen only in those with multi-morbidity (IRR 1.96; 95% CI 1.03, 3.72) and not in those with one (IRR 0.88; 95% CI 0.45, 1.71) or no morbidities (IRR 0.99; 95% CI 0.31, 3.23). Finally, we specifically looked at the effect of fatigue in the context of people reporting no pain, pain at a single site and pain at multiple sites. When separate, fully adjusted models were run, the effect of fatigue on HRJL was only present in those with multi-site pain (IRR 1.89; 95% CI 1.06, 3.38) and not in those with single site (IRR 0.63; 95% CI 0.15, 2.60) or no pain (IRR 0.45; 95% CI 0.11, 1.88). With fatigue analysed as a continuous variable, the crude IRR of HRJL increased by 1.08 (95% CI 1.06, 1.10) for every point increase in fatigue score while the fully adjusted IRR was 1.02 (95% CI 0.99, 1.04).

Do people with recent HRJL report higher levels of fatigue up to 1 year later, compared with those who have continued working? Amongst the 2722 participants included in this analysis, the median fatigue score assessed at 4 years follow-up was significantly higher amongst those with recent HRJL (i.e. those who experienced the event at any point between Follow-up 3 and Follow-up 4) (22 IQR [18, 27] versus 17 IQR [14, 21]) and 58% of those with HRJL satisfied criteria for fatigue, compared with 22% amongst those who continued working during that same period. Both on crude analysis (IRR 2.60; 95% CI 1.92, 3.51) and after full adjustment (IRR 1.91; 95% CI 1.39, 2.62), those with recent HRJL were significantly more likely to be fatigued a year later.

## Discussion

Fatigue was common amongst people working and was associated with markedly reduced productivity. Adjusting for differences in people with and without fatigue, high levels of fatigue independently predicted future job loss related to health, but only in those with multi-morbidity (including multi-site pain).

The HEAF study is well placed to examine factors associated with HRJL, although information on fatigue was only collected after the cohort was established and at a single point, and therefore, we are only able to examine, prospectively, the influence of fatigue over a short period. The factors influencing whether someone leaves employment can be complex, and such complexity is not captured in questionnaires. Further as three-quarters of the sample had a work impairment score of 0, we considered it a better approach to use it in its dichotomous form in the regression analysis. However, we appreciate that when continuous variables are dichotomized, information is lost. People with fatigue differ across several socio-economic and job-related factors, and several of these factors are related to HRJL. Reports from the HEAF study have previously shown that morbidities (including obesity), lack of ability to cope with the mental and physical demands of a job, and dissatisfaction with a job were all associated with HRJL [16–18]. In this analysis, full adjustment for socio-demographic, work-related and lifestyle factors suggested, however, that high levels of fatigue were independently associated with HRJL, specifically amongst those with multi-morbidity (including multi-site pain). We acknowledge that fatigue may have arisen from different pathophysiological processes such as fatigue as a co-morbidity to an inflammatory condition, fibromyalgia, chronic fatigue syndrome and post-viral syndrome. We do not have further information on the nature of the fatigue and any related diagnoses, but have shown that irrespective of the mechanisms by which it has arisen it has an important impact in relation to work.

Most research has concentrated on fatigue in the context of another morbidity, and the results from this study confirm that this is a reasonable approach in relation to HRJL. There is consistency in the literature on fatigue having a negative impact on productivity and risk of leaving employment. For example, we have shown in a national registry that, amongst people with axial spondyloarthritis, fatigue (along with a physically demanding job, poor function and high disease activity) is associated with lower work productivity, which itself predicts future absenteeism and subsequent leaving employment [19]. Intervening on fatigue (as an upstream factor), along with issues such as job modifications, may prevent these negative outcomes. Amongst cancer patients, it has been demonstrated that high levels of fatigue are associated with both early retirement and other non-employment [20]. Qualitative research emphasizes that fatigue (along with pain) is a key issue making it difficult to remain working or return to work/participating in society [21,22]. This is particularly due to the variable and unpredictable nature of these symptoms and strengthens the case for why programmes designed to support people to remain in work should include consideration of fatigue as an important component. Indeed, the Making it Work™ programme, developed by Arthritis Canada to support people with inflammatory arthritis to remain in work, includes a module on the self-management of fatigue [23]. Evaluation of the programme has shown that it reduced the likelihood of prolonged absence from work—although it is unclear which specific components of the programme have mediated this effect.

Assessing the effects of interventions for fatigue in relation to work outcomes is challenging because trials have not historically included work as a trial outcome. Individual trials would typically not have had sufficient power to demonstrate a significant change in work outcomes. Second, historically, outcomes have been chosen by researchers; as patient partners have become more involved in research studies, the importance of outcomes such as work participation has become evident. Our recent trial ‘Lessening the Impact of Fatigue in Inflammatory Rheumatic Diseases’ (LIFT) demonstrated that a personalized exercise programme resulted in a significant improvement in the severity and impact of fatigue (primary outcomes) and a significant improvement in work productivity (a secondary outcome). The effect on work productivity was the equivalent of people working full time having, on average, 1 day fewer per week at work impacted by fatigue [24]. This provides stronger evidence that there is a causal relationship between fatigue and work outcomes. A Cochrane review in relation to IBD found some evidence (but very uncertain) for both non-pharmacological (physical activity) and pharmacological (adalimumab) treatment effects on fatigue related to IBD [25], while Xu *et al*. [26] reported in a systematic review of 34 randomized controlled trials that cognitive behaviour therapy for persons on sick leave was associated with a lower total duration of sick leave and a return to work on average 1.5 days earlier. The study reported that CBT-based interventions ‘were effective in managing fatigue, mental illness, and depression, and improving physical function while they showed no effects in managing stress, anxiety and working ability’. The same conclusion on the effectiveness of CBT approaches for return to work was reached by a systematic review conducted as part of the CHRODIS Plus Joint European Action Project—it found that ‘coaching’ is effective in supporting the self-management of long-term conditions and is associated with a significant improvement in work capacity and perception of fatigue [27].

The current analysis adds to the body of evidence suggesting that addressing fatigue is likely to be important in supporting people to remain in, or return to, work. There are a variety of evidenced-based approaches including self-management, a personalized exercise programme, or cognitive behaviour therapy. The association observed in quantitative studies between fatigue and HRJL, the identification by patients that fatigue is a key factor in decisions to leave work, and the evidence that interventions addressing fatigue improve work outcomes, all provide evidence for a likely causative effect. It provides compelling evidence that support to manage fatigue is an important component to include in programmes for people with health conditions to remain in work.
